# Alterations in tryptophan metabolism and *de novo* NAD^+^ biosynthesis within the microbiota-gut-brain axis in chronic intestinal inflammation

**DOI:** 10.3389/fmed.2024.1379335

**Published:** 2024-07-02

**Authors:** Jeannie Devereaux, Ainsley M. Robinson, Rhian Stavely, Majid Davidson, Narges Dargahi, Ramya Ephraim, Dimitros Kiatos, Vasso Apostolopoulos, Kulmira Nurgali

**Affiliations:** ^1^Institute for Health and Sport, Victoria University, Melbourne, VIC, Australia; ^2^School of Rural Health, La Trobe University, Melbourne, VIC, Australia; ^3^Department of Medicine, Western Health, Faculty of Medicine, Dentistry and Health Sciences, The University of Melbourne, Melbourne, VIC, Australia; ^4^Department of Pediatric Surgery, Pediatric Surgery Research Laboratories, Massachusetts General Hospital, Harvard Medical School, Boston, MA, United States; ^5^Immunology Program, Australian Institute of Musculoskeletal Science (AIMSS), Melbourne, VIC, Australia; ^6^Regenerative Medicine and Stem Cells Program, Australian Institute of Musculoskeletal Science (AIMSS), Melbourne, VIC, Australia

**Keywords:** chronic intestinal inflammation, inflammatory bowel disease, colitis, gut-brain axis, tryptophan metabolism, nicotinamide metabolism, microbiota

## Abstract

**Background:**

Inflammatory bowel disease is an incurable and idiopathic disease characterized by recurrent gastrointestinal tract inflammation. Tryptophan metabolism in mammalian cells and some gut microbes comprise intricate chemical networks facilitated by catalytic enzymes that affect the downstream metabolic pathways of *de novo* nicotinamide adenine dinucleotide (NAD^+^) synthesis. It is hypothesized that a correlation exists between tryptophan *de novo* NAD^+^ synthesis and chronic intestinal inflammation.

**Methods:**

Transcriptome analysis was performed using high-throughput sequencing of mRNA extracted from the distal colon and brain tissue of *Winnie* mice with spontaneous chronic colitis and C57BL/6 littermates. Metabolites were assessed using ultra-fast liquid chromatography to determine differences in concentrations of tryptophan metabolites. To evaluate the relative abundance of gut microbial genera involved in tryptophan and nicotinamide metabolism, we performed 16S rRNA gene amplicon sequencing of fecal samples from C57BL/6 and *Winnie* mice.

**Results:**

Tryptophan and nicotinamide metabolism-associated gene expression was altered in distal colons and brains of *Winnie* mice with chronic intestinal inflammation. Changes in these metabolic pathways were reflected by increases in colon tryptophan metabolites and decreases in brain tryptophan metabolites in *Winnie* mice. Furthermore, dysbiosis of gut microbiota involved in tryptophan and nicotinamide metabolism was evident in fecal samples from *Winnie* mice. Our findings shed light on the physiological alterations in tryptophan metabolism, specifically, its diversion from the serotonergic pathway toward the kynurenine pathway and consequential effects on *de novo* NAD^+^ synthesis in chronic intestinal inflammation.

**Conclusion:**

The results of this study reveal differential expression of tryptophan and nicotinamide metabolism-associated genes in the distal colon and brain in *Winnie* mice with chronic intestinal inflammation. These data provide evidence supporting the role of tryptophan metabolism and *de novo* NAD^+^ synthesis in IBD pathophysiology.

## 1 Introduction

Accumulating evidence has shown that disruption of the gut-brain axis, a bidirectional communication system that involves neural, hormonal, metabolic, immunological, and microbial signals, may be related to the occurrence and development of gastrointestinal (GI) disorders, such as inflammatory bowel disease (IBD) (1). IBD is a chronic inflammatory disorder of the GI tract that can manifest as Crohn's disease (CD) or ulcerative colitis (UC) (2). Typical clinical manifestations of IBD include diarrhea, abdominal pain, weight loss, fatigue, tenesmus, perianal fissures, and bloody stool (2, 3). Progression of the disease may lead to complications, including fistulas, strictures, rectal bleeding, abscesses, bowel perforations, toxic megacolon, and an increased risk of colorectal cancer (4–6). Additionally, IBD is often complicated by psychological comorbidities, such as anxiety and depression, which affect patient quality of life and disease management (7). It is widely considered that the gut-brain axis disruption is a key element underlying the association between psychological distress and IBD (8, 9). While the etiology of IBD is yet to be fully elucidated, it is considered to be multifactorial, involving complex interactions between host genetic susceptibility, environmental exposures, gut dysbiosis, aberrant immune responses, and enteric nervous system (ENS) dysfunction (10).

IBD symptoms have been associated with dysregulation of 5-hydroxytryptamine (5-HT, serotonin), a tryptophan derivative, which is an essential amino acid and vital ENS signaling molecule (11, 12). 5-HT regulates energy metabolism, which is necessary for maintaining gut homeostasis (13, 14). In response to numerous stimuli, including nutrients, toxins, or neuromodulatory agents, enterochromaffin cells release 5-HT, which regulates gut motility, glucose absorption, and fluid balance (11). Recent evidence indicates that peripheral 5-HT is involved in multiple metabolic control pathways (13), including links to the brain relating to behavioral changes, anxiety, and depression (15). Intestinal tryptophan metabolism involves complex interactions between host genetic, microbial, and dietary factors. Tryptophan metabolites are produced via three major metabolic pathways: kynurenine, serotonin, and indole pathways, which have essential roles in the regulation of intestinal inflammation by acting directly or indirectly on pro/anti-inflammatory cytokines, immune cell function, intestinal microbial composition, and gut homeostasis (16). Results of studies investigating the connection between the gut-brain axis and intestinal inflammation suggest that dysregulated tryptophan metabolism contributes to disease severity and depressive symptoms in IBD patients and experimental models of colitis; however, these studies have limited their focus to only a few tryptophan metabolites (15, 17–20).

Gut microbial dysbiosis has been implicated in the pathogenesis of IBD and numerous studies mention it as a contributory factor for the development and progression of the disease (21, 22). Commensal gut microbes have diverse impacts on host tryptophan availability, directly and indirectly controlling tryptophan metabolism and contributing to the synthesis of neuroactive molecules, such as 5-HT, kynurenines, tryptamine, and indolic compounds which participate in the microbiota-gut-brain communication (23–25). Dysbiosis can be associated with imbalanced activation of the three pathways of tryptophan metabolism (26, 27). Furthermore, variations in tryptophan metabolism can negatively influence microbial proliferation and diversity; thus, the gut microbiota is considered a driving force affecting the tryptophan metabolism in the gut (24).

Despite the association between chronic intestinal inflammation and dysregulated tryptophan metabolism, few studies have investigated the intrinsic molecules involved in tryptophan metabolism that may distinguish the downstream physiological alterations in IBD. Approximately 90%−95% of tryptophan degradation occurs through the kynurenine pathway leading to *de novo* NAD^+^ biosynthesis (28), which depends on expressions of interferon (IFN)-γ, tumor necrosis factor (TNF)-α, bacterial lipopolysaccharides, and the pro-inflammatory cytokine interleukin (IL)-6, all of which are key pathogenic factors in IBD (28). Deviations from NAD^+^ homeostasis distort NAD-dependent enzyme levels and impact DNA repair, neuron regeneration, and inflammation resistance, contributing to the development of a spectrum of diseases (29). However, studies investigating NAD^+^ biosynthesis have been restricted to the comparison of just one or two genes within these pathways (30). Indole, or the aryl hydrocarbon receptor (AhR) pathway, metabolizes 4%−6% of tryptophan into indole derivatives via gut microbiota, including *Lactobacillus, Bifidobacterium, Clostridium*, and *Bacteroides*, activating AhR, which is important in the modulation of intestinal inflammation (24, 31–33). About 1%−2% of tryptophan enters the serotonergic pathway, which processes tryptophan mainly via tryptophan hydroxylase 1 (TPH1) within enterochromaffin cells in the gut or TPH2 in the brain into 5-HT and melatonin (34). Enterochromaffin cell hyperplasia and downregulation of serotonin reuptake transporter lead to elevated levels of 5-HT within the colonic mucosa, contributing to the onset and progression of chronic intestinal inflammation, shown in the *Winnie* mouse model of chronic colitis (35).

*Winnie* mice, an experimental model of spontaneous chronic colitis, are affected by a missense mutation of the *Muc2* gene, resulting in aberrant biosynthesis of mucin within the goblet cells (36). Consequently, progressive chronic intestinal inflammation develops, with signs appearing from 6 weeks of age, progressing to severe colitis by 12–16 weeks of age (37). Although previous studies have demonstrated inflammation-induced structural and functional alterations in the *Winnie* mouse colon, establishing this experimental model to be highly representative of IBD (36–42), no studies have evaluated tryptophan metabolism and *de novo* NAD^+^ biosynthesis in *Winnie* mice. Since IBD development is closely related to gene interactions within diseased tissue (43), this study aimed to determine changes in the tryptophan *de novo* NAD^+^ synthesis. Subsequently, we aimed to investigate correlation between dysregulated tryptophan metabolism in the colon and the brain and fecal microbiota involved in tryptophan metabolism using the *Winnie* mouse model. Enhancing understanding of changes in the tryptophan *de novo* NAD^+^ synthesis may potentially reveal novel insights into IBD pathophysiology.

## 2 Methods

### 2.1 Animals

*Winnie* (males and females, aged 14 weeks, *n* = 17) and C57BL/6 mice (males and females, aged 14 weeks, *n* = 18) were obtained from the same breeding colony at the Victoria University Werribee Animal Facility (Melbourne, Victoria, Australia) and delivered to the Animal Holding Facility at the Western Centre for Health, Research and Education (Melbourne, Victoria, Australia), where experiments were performed. All mice were permitted *ad libitum* access to food and water and housed in a temperature-controlled environment with a 12-h day/night cycle. Animals were acclimatized for 1 week prior to euthanasia via administration of pentobarbitone (1:16 dilution, 30 g, 100 μl/20 g, Lethabarb, Virbac, Australia). The distal colon tissues and brains were excised and used for subsequent experiments. All experimental procedures adhered to the Australian National Health and Medical Research Council (NHMRC) guidelines and were approved by the Victoria University Animal Experimentation Ethics Committee (AEC-17-016).

### 2.2 RNA extraction, quality control, and high-throughput RNA sequencing of distal colon and brain samples

Total RNA was extracted from the distal colons and brains of C57BL/6 (*n* = 8) and *Winnie* (*n* = 6) mice using the RNeasy Lipid Tissue Mini Kit (Qiagen, Hilden, Germany), as per manufacturer instructions. The extracted RNA underwent a quality assessment on a 2100 Bioanalyzer using the RNA 6000 Nano Kit (Agilent Technologies, USA) to confirm that the samples were devoid of genomic DNA contamination and 16S ribosomal RNA from bacteria. RNA concentration was then measured by a Nanodrop spectrophotometer (Denovix, Melbourne, Australia), with 260/280 nm and 260/230 nm ratios exceeding 1.8, indicating purity. The mRNA samples were submitted to Micromon Genomics (Monash University, Melbourne, Australia) for RNA-sequencing (RNA-Seq) using the next-generation sequencing (NGS) platform. Micromon Genomics performed RNA quality control with Invitrogen Qubit RNA BR assay (Gene Target Solutions, Sydney, Australia), as well as the polyA purification of mRNA from total RNA samples and RNA-Seq library construction. All samples quantified and analyzed with the Qubit and Bio-analyzer passed the QNA quality control (QC) standard. Micromon Genomics conducted the bioinformatics and performed the sequencing on MGITech MGISEQ2000RS hardware (MGISEQ-2000RS High-throughput Sequencing Set). The MGIEasy V2 chemistry set was used to generate the library, with at least 400 m raw reads per lane and paired-end 100b reads for accuracy and processing to compressed FASTQ files. Raw FASTQ files were analyzed using the RNAsik pipeline version 1.5.4 (44) and the *Mus musculus* reference genome GRCm38 (GenBank accession GCA_000001635.2) (45). Raw read counts were quantified using the FeatureCounts program (46) and analyzed with Degust for counts per million (CPM) library size normalization (47). Differentially expressed genes (DEGs) of the colon and brain were identified using EdgeR with a false discovery rate (FDR) of < 0.001, applying the Benjamini–Hochberg correction (48). Genes with log_2_ fold change (FC) value of ±0.585 (FC ± 1.5) were selected as the cut-off. To analyze differential gene expression based on overrepresentation analysis, software for the Kyoto Encyclopedia of Genes and Genomes (KEGG) pathways was used (49). Sequencing data are deposited at the Gene Expression Omnibus (GEO) repository, with the accession numbers GSE244558 (colon) and GSE264317 (brain).

### 2.3 Data visualization for tryptophan and nicotinamide metabolism associated gene expressions

Gene lists for tryptophan and nicotinamide metabolism-associated gene expressions were generated by merging related gene lists from multiple databases accessed via the molecular signature database (MSigDB). Heat maps of tryptophan and nicotinamide metabolism-associated gene expression values for each colon sample were visualized using the Morpheus web-based tool and presented as *Z*-score distributions (across samples) of the CPM values. Data are hierarchically clustered by Euclidian distance. Visualization of selected pathways was performed using the R package, Pathview, using default parameters (50). Input data included significance scores of gene expression values between *Winnie* and C57BL/6 mice computed as the sign Log_2_FC × Log_10_*P* and represented in a KEGG graph.

### 2.4 Homology of genes in *Winnie* mice and IBD patients in colonic tryptophan and nicotinamide metabolism associated genes

The homology of genes in *Winnie* mice and IBD patients was determined as previously described (51). Briefly, the National Center for Biotechnology Information (NCBI) GEO public data source was used to gather gene expression data on the transcriptome of human IBD patients (52, 53) to establish homology to tryptophan and nicotinamide metabolism DEGs identified in the *Winnie* mouse colon in this study. High-throughput sequencing was used to create the expression profile of the inflamed intestine in IBD patients and the healthy colon regions in uninflamed controls undergoing resection of non-obstructive colorectal adenocarcinoma. Peters et al. (54) assembled these data as reads per kilobase of transcript per million mapped reads. Similar to our methods, polyA-purified mRNA was used in this study from RNA isolated using TRIzol. These data are available at https://www.ncbi.nlm.nih.gov/geo/ under the GEO series accession number GSE83687. This dataset included CD patients (*n* = 42), UC patients (*n* = 31), and controls (*n* = 60).

### 2.5 Preparation of standards for measuring tryptophan metabolite concentrations

Reference standards: l-tryptophan; 5-hydroxyindole-acetic acid (5-HIAA), kynurenic acid (KYNA), l-kynurenine 98%, 2,3-pyridinedicarboxylic acid [quinolinic acid (QUIN)], nicotinamide, and 2-picolinic acid (PIC; all purchased from Sigma–Aldrich, Castle Hill, Australia) were accurately weighed using the calculation of normality and subsequently dissolved individually into the mobile phase composition (15% acetonitrile, 0.2% trifluoroacetic acid (TFA), and 84.8% Milli-Q purified water) degassed under vacuum. The eluents were filtered through a Millipore Membrane Filter (type HA, pore size 0.45 μm, Billerica, MA, USA); 0.5 ml of eluent was aliquoted into a 2 ml dark-colored glass bottle and injected into the ultra-fast liquid chromatograph (UFLC) for method validation (55, 56).

### 2.6 Chromatographic conditions

The Shimadzu Prominence UFLC (Shimadzu Corporation, Kyoto, Japan) consisted of variable wavelength UV-visible (UV-Vis) detectors. The high-precision six-port valve achieves 0.3% or less area repeatability in 100,000 cycle endurance tests. The measurements for simultaneous tryptophan compounds were obtained via the isocratic, ion-pair, reversed-phased C18 (250 mm length × 4.6 mm internal diameter) analytical column in the aforementioned mobile phase composition. The chromatogram was run under multistep gradient conditions with a pump flow rate of 0.8 ml/min, a column temperature of 25°C and an injection volume of 10 μl/min. The observed analysis column pump pressure fluctuated around 2500 pounds per square inch (psi). Elution was monitored by the photodiode array detector (collecting UV–Vis spectra from 190 to 800 nm). Standard retention times were monitored using the 254 nm wavelength.

### 2.7 Tryptophan metabolite standard solutions calibration curve and validation

The UFLC analytical method for measuring tryptophan metabolite concentration was validated for the parameters of selectivity, specificity, linearity, precision (robustness and ruggedness), accuracy (% recovery), sensitivity, limits of detection (LOD), and limits of quantification (LOQ) in calibrating the solutions. The linearity graph was plotted as ‘concentration vs. peak area response' via the Lab Solutions calibration curve function, with a correlation coefficient ranging from 0.9995 to 1.000 for all compounds. The intra-day reproducibility was assessed using five solutions daily, while the inter-day reproducibility was determined by measuring the same solutions on three consecutive days. Intra-day accuracy and precision were calculated from the % bias [% (measured–theoretical)/theoretical concentration] and relative standard deviation (RSD) [%RSD = % standard deviation/mean] for the five replicates of each QC point. LOD and LOQ values were calculated by expressing the noise-to-signal ratio of the lowest known concentration of linearity samples in μg/ml or ppm, then converted to percentages (mg/L). Following analysis, the output chromatogram displayed the constituents of standard solutions as a peak against the retention time. The retention times were utilized for quantitative analysis and detection of unknown concentrations in the biological samples (57). Our method is consistent with the profiles observed in preceding studies involving tryptophan metabolites (58, 59).

### 2.8 Preparation of distal colon and brain samples for UFLC

Biological samples of the distal colon and brains from C57BL/6 (*n* = 5–8) and *Winnie* mice (*n* = 6–8) were weighed and placed into safe-lock tubes, containing ice-cold 0.2 M HCIO_4_ solution (10 μl/mg of tissue). Homogenization of the tissues was performed in the pre-cooled homogenizing bead beater (TissueLyser LT, Qiagen, Melbourne, Australia). The homogenates were retained at 5°C for 15 min to aid the precipitation of proteins, cell debris, and microscopic tissues. Next, the homogenates were centrifuged at 12,000 revolutions per minute at 4°C for 15 min to extract the metabolites from the matrix. The supernatants were immediately separated from the precipitates and transferred into a sterile microtube, adjusted to a ratio of 4:1 v/v with ice-cold 0.1 M HClO_4_, vortexed for 30 s, and filtered into a new tube for subsequent experiments (60).

### 2.9 Analysis of tryptophan metabolites

Prepared distal colon and brain samples from C57BL/6 and *Winnie* mice were injected separately into the UFLC. The concentrations of each tryptophan metabolite were determined by analyzing the peak heights of individual analytes using the inbuilt Shimadzu software. The metabolite concentrations recorded in the Shimadzu acquired “American Standard Code for Information Interchange” text file analyzed by Origin Pro, Version 2021b (OriginLab Corporation, Northampton, MA, USA). All peak fitting, surface fitting, and signal processing capacities reached chi-square tolerance values of 1E-6.

### 2.10 Fecal microbiome analysis

Fecal samples were collected from C57BL/6 and *Winnie* mice (*n* = 11 for both) and stored at −80°C until microbial DNA extraction. Extraction of DNA was performed as previously described (40). Briefly, the MoBio PowerFecal DNA Isolation Kit (GeneWorks, Thebarton, South Australia) was used to extract DNA from 0.25 g of each fecal sample according to the manufacturer's instructions. Homogenization and lysis of cells were performed with a FastPrep-24 instrument (MP Biomedicals, Seven Hills, NSW, Australia). The DNA was cleaned with an ethanol-based wash solution to remove residual salt and other contaminants. Subsequently, a sterile elution buffer (10 mM Tris) released the DNA from the spin column filter, yielding DNA that was ready for downstream applications. Centrifugation steps were performed for 1 min at 13,000 g. PCR amplicons spanning the 16S rRNA V3–V4 hypervariable region (forward primer: CCTAYGGGRBGCASCAG, reverse primer GGACTACNNGGGTATCTAAT) underwent high-throughput sequencing on the Illumina MiSeq platform using a 300 bp paired end protocol (600 cycles, 0.04–0.10 GB per run) at the Australian Genome Research Facility (University of Queensland, Brisbane, Australia). All microbiota data have been uploaded to the GitHub repository. The data are accessible via https://github.com/Nurgali-lab/C57-Win-APX-16SV3-4.git. Bioinformatics analysis involved demultiplexing, QC, Amplicon Sequence Variant (ASV) calling, and taxonomic classification. Briefly, diversity profiling analysis was performed with QIIME 2 2019.7. The demultiplexed raw reads were primer trimmed and quality filtered using the cutadapt plugin followed by denoising with DADA2 (via q2-dada2). Taxonomy was assigned to ASVs using the q2-featureclassifier classify-sklearn naïve Bayes taxonomy classifier. Evaluations present at the genus taxonomic level, including percentage compilations, represent all sequences resolved to their primary identification or their closest relative. The relative abundance of genera in fecal samples from C57BL/6 and *Winnie* mice was calculated by the number of ASVs relative to the total number of ASVs per sample and means compared between groups in this study. Genera involved in tryptophan and nicotinamide metabolism were identified from previous reports, including some studies in IBD and colitis (23, 24, 61–67). Alpha diversity was performed for species richness, Chao1, Shannon–Wiener's diversity index and Simpson's index. Beta diversity was obtained by calculating the Bray-Curtis dissimilarity index. Calculations were performed using Microsoft Excel.

### 2.11 Statistical analysis

GraphPad Prism v9 (GraphPad Software, San Diego, CA, USA) was used to determine statistical differences using an unpaired *t*-test with Welch's correction. Data are presented as the mean ± standard error of the mean (SEM). Differences were considered statistically significant when *p* < 0.05.

## 3 Results

### 3.1 Changes in tryptophan and nicotinamide metabolism associated gene expression in the *Winnie* mouse colon

#### 3.1.1 Genes and pathways related to tryptophan and nicotinamide metabolism were altered in the *Winnie* mouse colon

**Figure 1:**
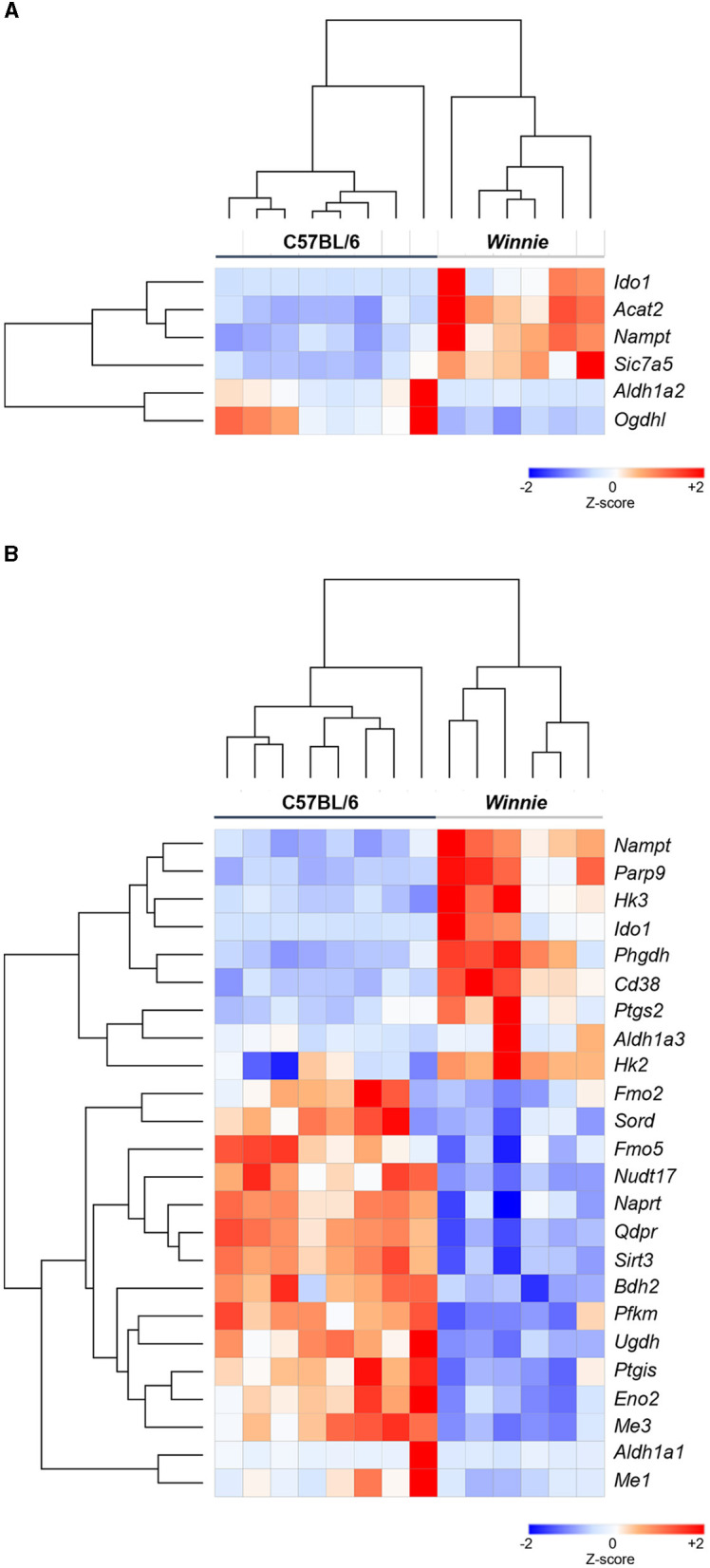
Heatmap representation of DEGs associated with tryptophan and nicotinamide metabolism in the *Winnie* mouse colon Heat map representations of tryptophan **(A)** and nicotinamide **(B)** metabolism-associated DEGs in colons from *Winnie* mice (*n* = 6) compared to colons from C57BL/6 mice (*n* = 8). Individual colon samples from C57BL/6 and *Winnie* mice are visualized in columns and DEGs are represented in rows. Plotted data presented as *Z*-score distributions (−2 to +2 across samples) of the counts per million values indicate gene expression values from DEGs analysis. Red represents upregulated gene expression, while blue represents downregulated gene expression.

Gene lists for tryptophan and nicotinamide metabolism-associated gene expressions were generated by merging related gene lists from multiple databases accessed via the MSigDB (Supplementary Figure S1). Subsequently, RNA-Seq was performed to determine changes in these gene expressions in the distal colon of *Winnie* mice (*n* = 6) vs. age-matched C57BL/6 littermates (*n* = 8). Heat maps were used to visualize DEGs associated with tryptophan and nicotinamide metabolism and are shown as individual sample *Z*-scores from the colons of C57BL/6 and *Winnie* mice (Figure 1). In total, six DEGs associated with tryptophan metabolism were observed in colons from *Winnie* when compared to samples from C57BL/6 mice (Figure 1A), while 24 DEGs associated with nicotinamide metabolism were noted (Figure 1B).

**Figure 2:**
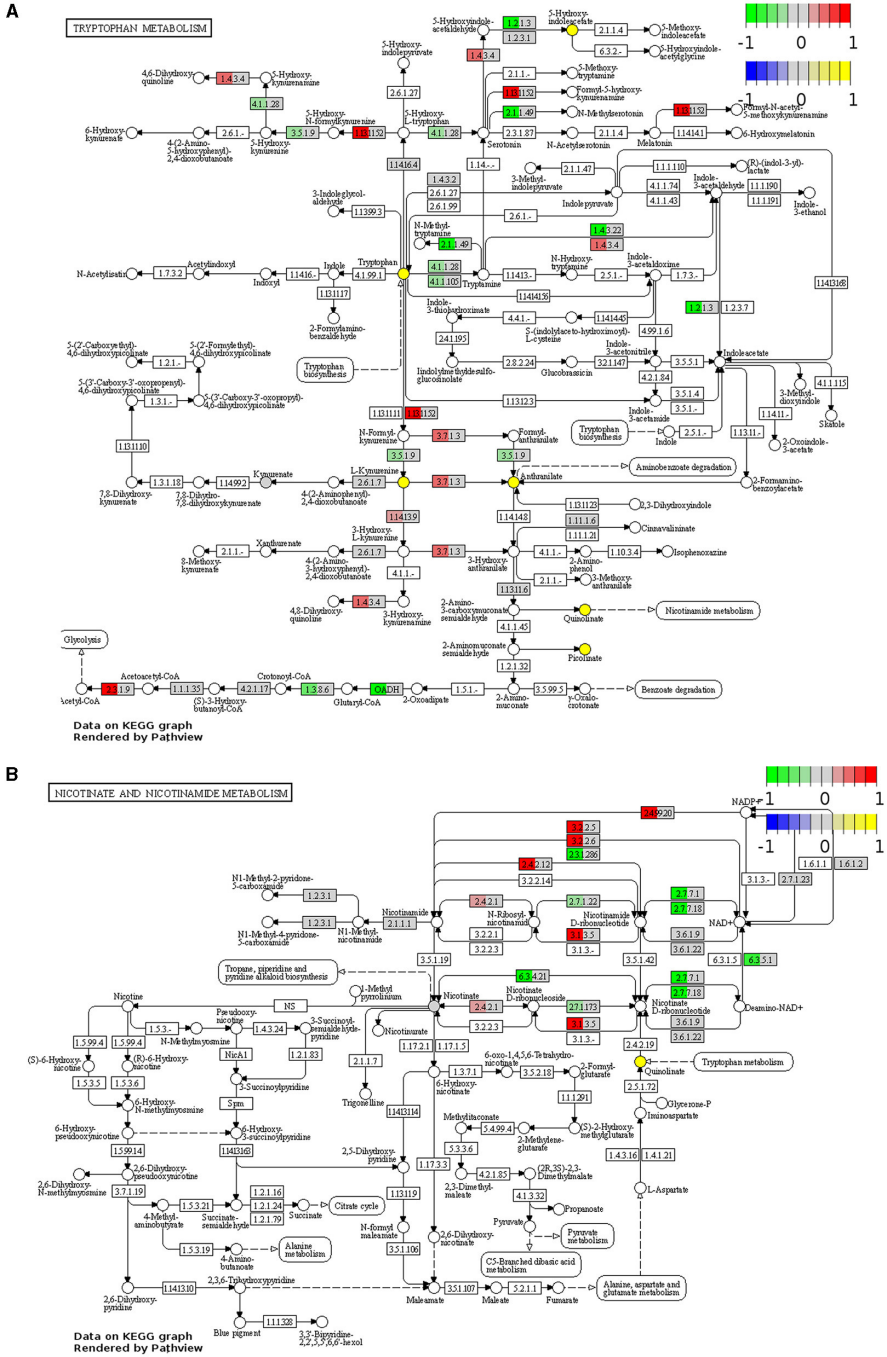
Functional analysis identifies upregulation and downregulation of key elements of tryptophan and nicotinamide metabolism in the *Winnie* mouse colon. Tryptophan **(A)** and nicotinamide **(B)** metabolism KEGG pathway analysis of distal colon samples from *Winnie* mice (*n* = 6) vs. C57BL/6 littermates (*n* = 8). DEGs of the tryptophan and nicotinamide metabolism KEGG pathways are colored according to their sign Log_2_FC × Log_10_*P* value changes. Rectangular nodes indicate gene expression data by RNA-seq gene expression data. Positive values (red) indicate genes that are upregulated (tryptophan—EC:1.4.3.4, *Mao-b*; EC:1.13.11.52, *Ido1*; EC:3.7.1.3, *Kynu*; EC:2.3.1.9, *Acat1*, nicotinamide—EC:2.4.99.20, *Cd38*; EC:3.2.2.6, *Cd38*; EC:3.2.2.5, *Art2a*; EC:2.4.2.12, *Nampt1*; EC:3.1.3.5, *Cd73*; EC:2.4.2.1, *Pnp*) and negative values (green) indicate genes that are downregulated [tryptophan—(EC):3.5.1.9, *Afmid*; EC:4.1.1.28 and EC:4.1.1.105, *Ddc*; EC:2.1.1.49, *SAMe*; EC:1.4.3.22, *Aoc1*; EC:1.2.1.3, *Aldh2*; OADH, *Dhtkd1*; EC:1.3.8.6, *Gcdh*, nicotinamide—EC:2.3.1.286, *Sirt2*; EC:2.7.7.1, *Nmnat2*; EC:2.7.7.18, *Nmnat3*; EC:2.7.1.22, *Nmrk1*; EC:2.7.1.173, *Nmrk2*; EC:6.3.5.1, *Nadsyn1*; EC:6.3.4.21, *Naprt*] in the *Winnie* mouse colon relative to colons from C57BL/6 mice.

Visualization of the KEGG tryptophan and nicotinamide metabolism pathways underscored changes to the regulation of key enzymes involved in these pathways in the *Winnie* mouse colon compared to colon samples from C57BL/6 mice (Figure 2). In colons from *Winnie* mice, analysis revealed a total of 11 DEGs involved in the tryptophan metabolic pathway (Figure 2A). In the *Winnie* mouse colon, the expression of genes involved in the catabolic process of tryptophan to kynurenine and the *de novo* NAD^+^ biosynthesis [kynureninase (*Kynu*), indoleamine 2,3-dioxygenase1 (*Ido1*)] were upregulated. Increased expression of *Kynu, Ido1*, and acetyl-CoA acetyltransferase 1 (*Acat1*) is associated with driving cellular amino acid catabolic processes and nucleotide biosynthetic processes. Additionally, upregulation of *Kynu* and *Acat1* implicate the acetyl-CoA metabolic process, potentially indicating heightened energy metabolism. An increase in the expression of *Kynu, Ido1, Acat1*, and monoamine oxidase-b (*Mao-b*) suggests enhanced cellular catabolic processes, while downregulation in glutaryl-CoA dehydrogenase (*Gcdh*) and aryl-formamidase (*Afmid*) suggests reduced substrate availability for tryptophan metabolism. There was a substantial reduction in the genes involved in the cellular metabolism of biogenic amines, indole-containing compounds, and cellular amino acid metabolic processes.

There were 12 DEGs associated with the nicotinamide metabolism pathway in the *Winnie* mouse colon when compared to colons from C57BL/6 mice (Figure 2B). Upregulation of ecto-5′-nucleotidase (*Cd73*) and purine-nucleoside phosphorylase (*Pnp*) suggests adenosine monophosphate (AMP) catabolic activity, adenosine metabolic processes, and the biosynthetic process for purine ribonucleosides. Nicotinamide phosphoribosyltransferase (*Nampt*) and *Pnp* upregulation can be associated with the metabolic processing of pyridine-containing compounds, while cluster of differentiation 38 (*Cd38*) plays a significant role in broader metabolic processes. The downregulation in nicotinamide ribose kinases (*Nmrk*), nicotinamide mononucleotide adenylyl transferases (*Nmnat*) nicotinamide adenine dinucleotide synthetase 1 (*Nadsyn1*), nicotinate phosphoribosyltransferase (*Naprt*), and silent information regulator 2 (*Sirt2*) are known to affect biological processes of NAD^+^ biosynthesis, catalytic activity, and adenosine triphosphate (ATP) binding.

#### 3.1.2 DEGs of tryptophan and nicotinamide metabolism in the *Winnie* mouse colon show homology to human IBD

**Figure 3:**
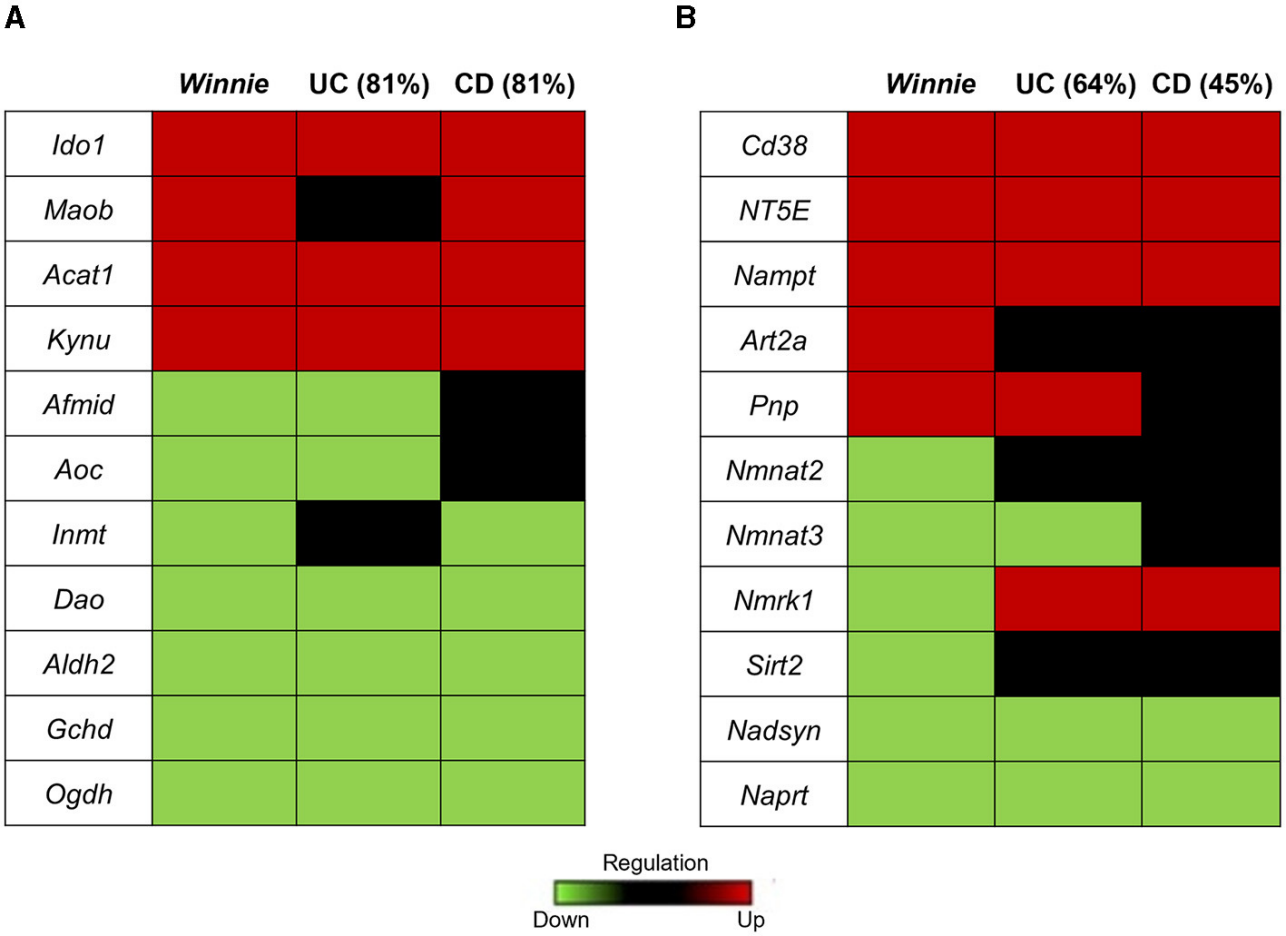
Homology of tryptophan and nicotinamide gene expression in colon samples from *Winnie* mice and IBD patients. Heat map representations of homology of tryptophan **(A)** and nicotinamide **(B)** metabolism associated genes in *Winnie* mice and IBD patients. Upregulated gene expression levels are marked red, low expression levels are marked green, and neutral expression levels are marked black.

The homology in the expression of target genes involved in tryptophan and nicotinamide metabolism between colon samples from *Winnie* mice and IBD patients was determined respectively to their uninflamed controls (Figure 3). We found 81% concordance in the expression of genes associated with tryptophan metabolism in *Winnie* mice with both UC and CD patients (Figure 3A). Evaluation of homology for nicotinamide metabolism-associated genes in *Winnie* mice colons revealed a 64% concordance with UC patients and 45% with CD patients (Figure 3B). These findings further underscore the relevance and significance of the *Winnie* mouse as a model for exploring the pathophysiology of IBD.

#### 3.1.3 Altered concentrations of tryptophan metabolites in the *Winnie* mouse colon

**Figure 4:**
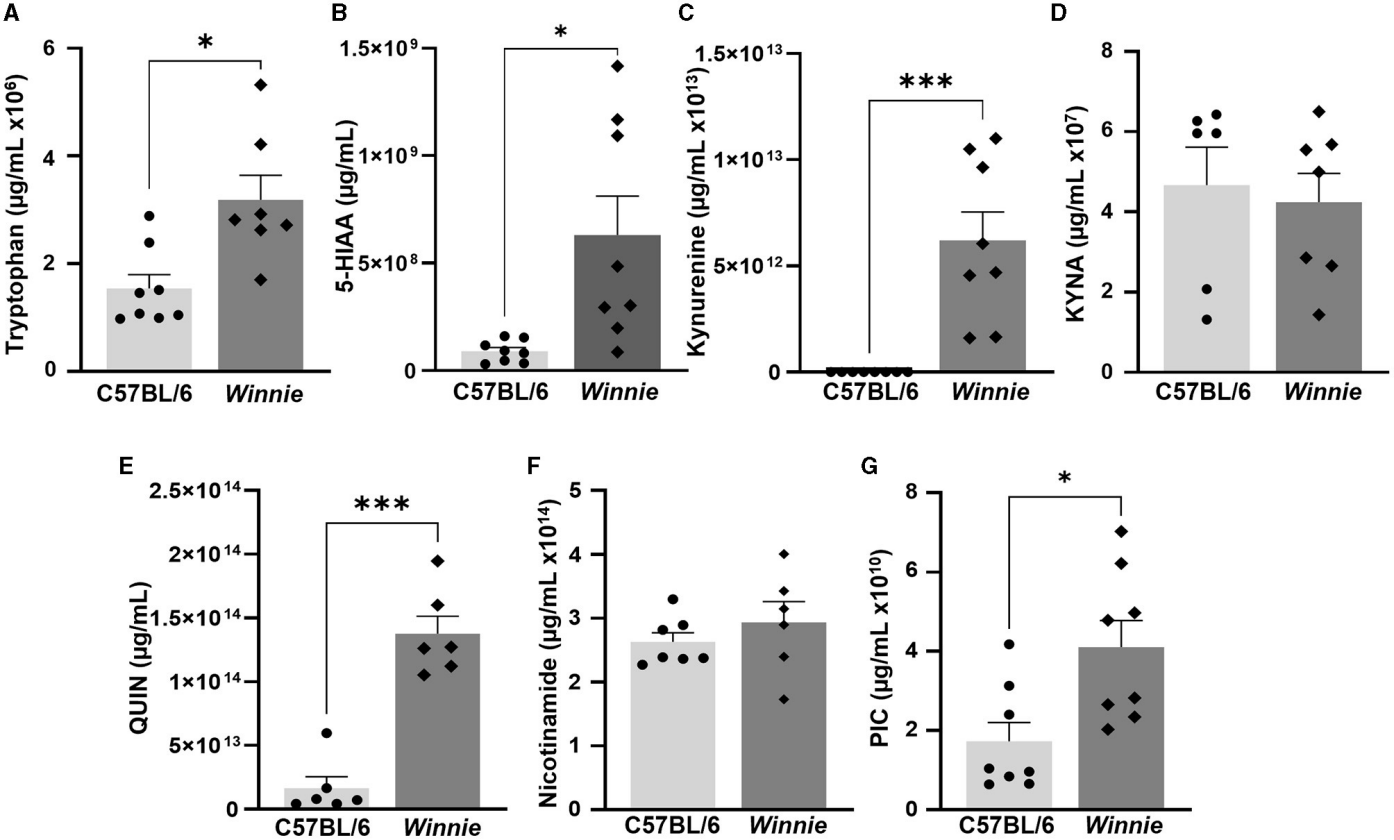
Changes in tryptophan metabolite concentrations in the *Winnie* mouse colon. Quantification of tryptophan metabolites (μg/ml) in *Winnie* mice distal colons compared to colons from C57BL/6 littermates (*n* = 6–8) as measured by UFLC: **(A)** Tryptophan, **(B)** 5-HIAA, **(C)** Kynurenine, **(D)** KYNA, **(E)** QUIN, **(F)** Nicotinamide, and **(G)** PIC. Data are expressed as mean ± SEM. *p < 0.05, ***p < 0.001 when compared to C57BL/6 mice.

Metabolomics analysis was employed to quantify tryptophan metabolites, including tryptophan, 5-HIAA, KYNA, kynurenine, QUIN, nicotinamide, and PIC, in the distal colons of *Winnie* mice and compared to colon samples from C57BL/6 littermates (*n* = 6–8 for both, Table 1, Figure 4). In *Winnie* mice colons, quantification of tryptophan metabolites revealed higher concentrations of tryptophan (*p* < 0.05), 5-HIAA (*p* < 0.05), kynurenine (*p* < 0.001), QUIN (*p* < 0.001), and PIC (*p* < 0.05) when compared to colons samples from C57BL/6 mice. No differences in concentrations of KYNA and nicotinamide were observed between groups (Table 1, Figure 4).

**Table 1:** Tryptophan metabolite concentrations in the colons from C57BL/6 and *Winnie* mice.

**Metabolite**	**Concentration (**μ**g/ml)**	
	**C57BL/6**	***Winnie***
Tryptophan	1.54 × 10^6^ ± 0.25 × 10^6^, *n* = 8	3.19 × 10^6^ ± 0.45 × 10^6^^*^, *n* = 7
5-hydroxyindole-acetic acid (5-HIAA)	0.90 × 10^8^ ± 0.18 × 10^8^, *n* = 8	6.31 × 10^8^ ± 1.81 × 10^8^^*^, *n* = 8
Kynurenine	5.65 × 10^7^ ± 2.28 × 10^7^, *n* = 8	6.21 × 10^12^ ± 1.34 × 10^12^^***^, *n* = 8
Kynurenic acid (KYNA)	4.67 × 10^7^ ± 0.95 × 10^7^, *n* = 6	4.24 × 10^7^ ± 0.72 × 10^7^, *n* = 7
Quinolinic acid (QUIN)	1.66 × 10^13^ ± 0.88 × 10^13^, *n* = 6	13.76 × 10^13^± 1.38 × 10^13^^***^, *n* = 6
Nicotinamide	2.63 × 10^14^ ± 0.14 × 10^14^, *n* = 7	2.94 × 10^14^ ± 0.33 × 10^14^, *n* = 6
Picolinic acid (PIC)	1.73 × 10^10^ ± 0.47 × 10^10^, *n* = 8	4.11 × 10^10^ ± 0.67 × 10^10^^*^, *n* = 8

^*^p < 0.05, ^***^p < 0.001 when compared to C57BL/6 mice.

### 3.2 Changes to the relative abundance of fecal microbial genera involved in tryptophan and nicotinamide metabolism in *Winnie* mice

**Figure 5:**
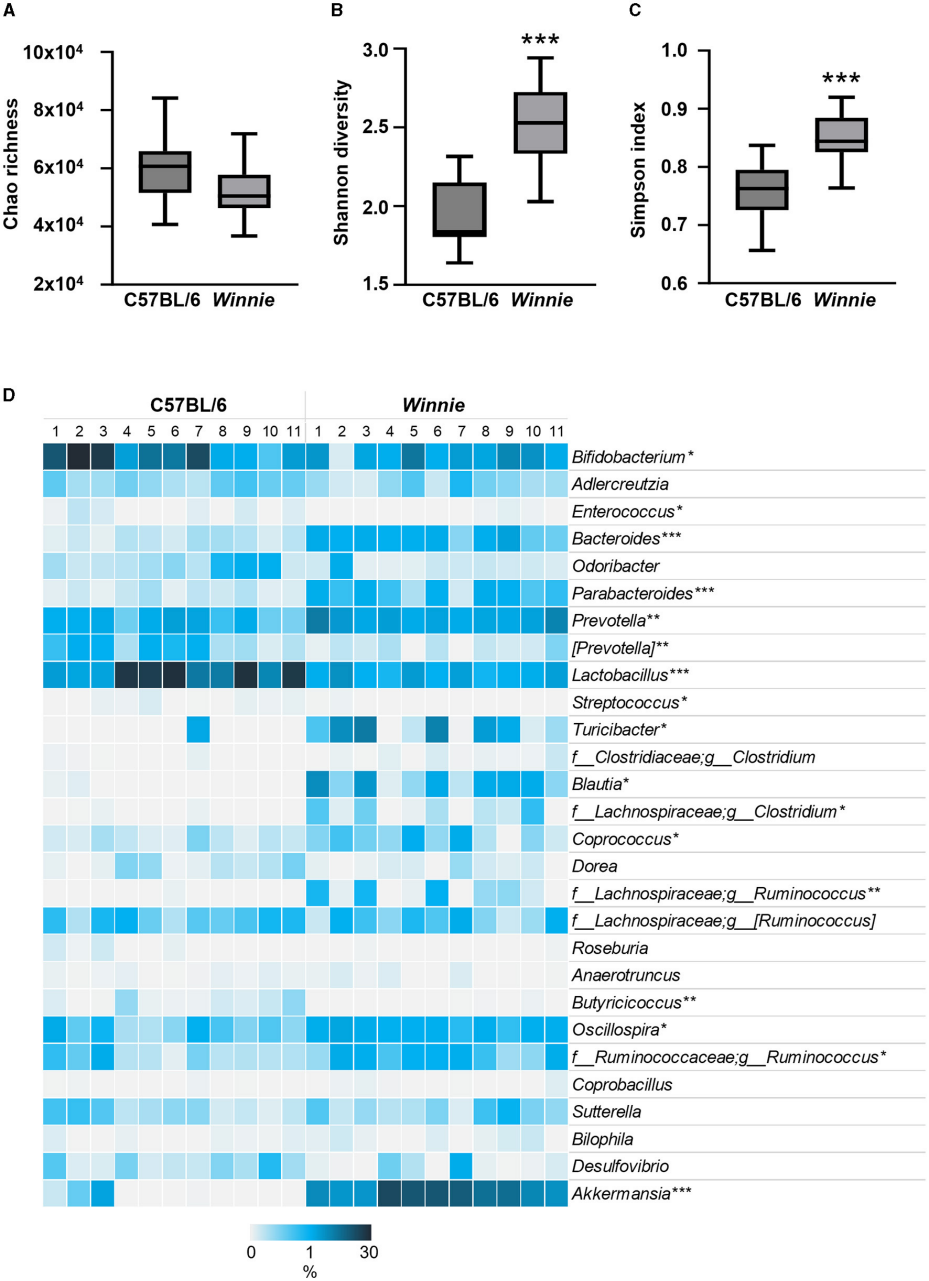
Changes to tryptophan and nicotinamide metabolism-associated microbiota at the genus level in fecal samples from *Winnie* mice. Microbiota communities were profiled from C57BL/6 and *Winnie* mice (*n* = 11/group). **(A)** Chao's richness estimate, **(B)** community diversity assessed by calculating the Shannon diversity, and **(C)** evenness by Simpson index based on abundance data. **(D)** Heat map representation of the percent relative abundance of tryptophan and nicotinamide metabolism-associated microbiota at the genus level in fecal samples from *Winnie* compared to C57BL/6 mice. Individual samples from C57BL/6 and *Winnie* mice are visualized in columns and genus level microbiota involved in tryptophan metabolism are represented by rows. Percent relative abundance was calculated by the number of ASVs by taxonomic classification at the genus level relative to the total number of ASVs per sample. Asterisks mark significant differences in the mean percent relative abundance between groups. *p<0.05, **p<0.01, ***p<0.001 when compared to C57BL/6 mice.

Since gut microbes contribute to the synthesis of neuroactive molecules and indolic compounds (23–25) and dysbiosis can be associated with disrupted tryptophan metabolism (26, 27), we next investigated whether changes in tryptophan metabolite concentrations in *Winnie* mouse colons corresponded to changes in the abundance of gut microbes involved in tryptophan and nicotinamide metabolism. To evaluate the relative abundance of gut microbial genera involved in tryptophan and nicotinamide metabolism, we performed 16S rRNA gene sequencing of fecal samples from C57BL/6 and *Winnie* mice (*n* = 11 for both). α-diversity was assessed based on community richness (Chao1), diversity (Shannon–Wiener diversity index), and evenness (Simpson index). Community richness (*p* = 0.06) did not differ significantly between groups, however, diversity and evenness were increased in *Winnie* (*p* < 0.001 for both) when compared to C57BL/6 mice (Figures 5A–C). For β-diversity metrics, the Bray-Curtis dissimilarity was calculated as 0.37, indicating a 37% difference in the relative abundance of genera between fecal samples from C57BL/6 and *Winnie* mice. At the genus level, the abundance of microbiota in samples from *Winnie* mice was distinctly different to samples from C57BL/6 mice, including substantial increases (FC > 10) in *Turicibacter*, unknown *Christensenellaceae*, unknown *Clostridiaceae, Blautia, Butyrivibrio, Clostridium* (*Lachnospiraceae* family), *Ruminococcus* (*Lachnospiraceae* family), unknown *Desulfovibrionaceae*, unknown *ML615J-28*, and *Akkermansia*, as well as decreased *Butyricicoccus* (Table 2). Since this study aimed to investigate whether there is a correlation between changes in tryptophan metabolites and changes in intestinal microbiota involved in tryptophan and nicotinamide metabolism in *Winnie* mice, we identified genus level tryptophan and nicotinamide metabolism-associated microbiota from previous studies, including studies in IBD and colitis (23, 24, 61–67). Analysis revealed dysbiosis of microbiota involved in tryptophan and nicotinamide metabolism in fecal samples from *Winnie* mice compared to samples from C57BL/6 mice (Table 2, Figure 5D). Key changes comprised large-scale decreases in the abundance of *Lactobacillus* (*p* < 0.001) and *Bifidobacterium* (*p* < 0.05), as well as increased abundance of *Akkermansia* (*p* < 0.001) in *Winnie* mice feces, demonstrating clear alterations in the composition of bacteria that contribute to the synthesis and/or degradation of tryptophan metabolites. We also observed increased abundance of certain indole-producing bacteria, including *Bacteroides* (*p* < 0.001), *Prevotella* (*p* < 0.01), *Ruminococcus* (*Ruminococcaceae* family) (*p* < 0.05), and *Oscillospira* (*p* < 0.05) in samples from *Winnie* mice, whereas the abundance of others, such as *Enterococcus* (*p* < 0.05), and *Butyricicoccus* (*p* < 0.01) decreased. Similarly, there were changes in the abundance of indole-3-lactic acid (ILA), indole-3-propionic acid (IPA), and indole-3-lactic acid (IAA) synthesizing bacteria in *Winnie* mice feces, including increases in *Parabacteroides* (*p* < 0.001) and *Coprococcus* (*p* < 0.05), while the abundance of *[Prevotella]* (*p* < 0.01) was reduced. *Streptococcus*, a 5-HT-producing microbe was decreased in samples from *Winnie* mice, while *Turicibacter*, a bacterium that directly imports 5-HT through a mechanism similar to a mammalian serotonin transporter, was significantly increased (*p* < 0.05 for both). Furthermore, the abundance of tryptamine-synthesizing *Blautia* (*p* < 0.05) and *Ruminococcus* (*Lachnospiraceae* family) (*p* < 0.01) were increased in samples from *Winnie* mice when compared to samples from C57BL/6 mice. These results highlight colitis-associated changes in tryptophan and nicotinamide metabolism in the microbiome; however, it is difficult to ascertain whether microbiota involved in these pathways are more affected than those involved in other pathways considering genera may have unknown functions participating in tryptophan metabolism that are yet to be reported in the literature.

**Table 2:** Relative abundance (%) of genera in fecal samples from C57BL/6 and *Winnie* mice.

**Genera**	**Relative abundance (%)**	
	**C57BL/6**	***Winnie***
*Bifidobacterium*	13.99 ± 3.82	5.19 ± 1.27^*^
*Coriobacteriaceae unknown*	0.24 ± 0.07	0.09 ± 0.02
*Adlercreutzia*	0.47 ± 0.05	0.40 ± 0.07
*Enterococcus*	0.06 ± 0.02	0.01 ± 0.01^*^
*Olsenella*	0.13 ± 0.04	0.11 ± 0.03
*Bacteroidales unknown*	0.14 ± 0.02	1.19 ± 0.26^**^
*Bacteroides*	0.20 ± 0.03	1.61 ± 0.34^***^
*Parabacteroides*	0.15 ± 0.03	1.03 ± 0.18^***^
*Prevotella*	1.83 ± 0.43	5.14 ± 1.07^**^
*Rikenellaceae unknown*	0.42 ± 0.06	0.81 ± 0.10^**^
*Rikenella*	0.05 ± 0.01	0.03 ± 0.01
*S24-7;g__*	31.58 ± 1.64	31.15 ± 1.84
*Odoribacter*	0.54 ± 0.15	0.30 ± 0.18
*[Prevotella]*	0.73 ± 0.15	0.18 ± 0.04^**^
*YS2 unknown*	1.32 ± 0.70	2.08 ± 0.78
*Mucispirillum*	0.02 ± 0.01	0.07 ± 0.01^*^
*Firmicutes unknown*	0.00 ± 0.00	0.02 ± 0.01
*Lactobacillus*	16.97 ± 3.22	2.90 ± 0.77^***^
*Streptococcus*	0.03 ± 0.01	0.00 ± 0.00^*^
*Turicibacter*	0.27 ± 0.27	3.81 ± 1.50^*^
*Clostridiales unknown*	1.97 ± 0.23	1.57 ± 0.28
*Clostridiales unknown*	2.57 ± 0.47	3.90 ± 0.52
*Christensenellaceae unknown*	0.01 ± 0.00	0.33 ± 0.08^**^
*Clostridiaceae unknown*	0.02 ± 0.02	1.46 ± 0.40^**^
*Candidatus Arthromitus*	0.04 ± 0.01	0.03 ± 0.02
*f__Clostridiaceae;g__Clostridium*	0.01 ± 0.01	0.05 ± 0.02
*Dehalobacterium*	0.10 ± 0.02	0.12 ± 0.03
*Lachnospiraceae unknown*	20.50 ± 5.21	7.79 ± 1.31^*^
*Blautia*	0.01 ± 0.01	2.38 ± 0.87^*^
*Butyrivibrio*	0.05 ± 0.03	1.07 ± 0.25^**^
*f__Lachnospiraceae;g__Clostridium*	0.01 ± 0.01	0.26 ± 0.09^*^
*Coprococcus*	0.20 ± 0.04	0.61 ± 0.17^*^
*Dorea*	0.21 ± 0.06	0.10 ± 0.03
*Roseburia*	0.03 ± 0.02	0.00 ± 0.00
*f__Lachnospiraceae;g__Ruminococcus*	0.00 ± 0.00	0.35 ± 0.12^**^
*f__Lachnospiraceae;g__[Ruminococcus]*	0.70 ± 0.08	0.78 ± 0.18
*Peptostreptococcaceae unknown*	0.00 ± 0.00	0.01 ± 0.00
*Ruminococcaceae unknown*	0.41 ± 0.08	2.51 ± 0.18^***^
*Anaerotruncus*	0.04 ± 0.01	0.04 ± 0.01
*Butyricicoccus*	0.13 ± 0.04	0.01 ± 0.00^**^
*Oscillospira*	0.78 ± 0.19	1.56 ± 0.22^*^
*f__Ruminococcaceae;g__Ruminococcus*	0.50 ± 0.16	1.07 ± 0.19^*^
*[Mogibacteriaceae] unknown*	0.05 ± 0.01	0.12 ± 0.02^**^
*Erysipelotrichaceae unknown __*	0.02 ± 0.01	0.00 ± 0.00^*^
*Allobaculum*	1.09 ± 0.37	2.05 ± 0.56
*Coprobacillus*	0.01 ± 0.00	0.02 ± 0.01
*RF32 unknown*	0.03 ± 0.01	0.10 ± 0.02^*^
*Sutterella*	0.38 ± 0.07	0.45 ± 0.08
*Desulfovibrionaceae unknown*	0.00 ± 0.00	0.18 ± 0.04^**^
*Bilophila*	0.03 ± 0.01	0.06 ± 0.02
*Desulfovibrio*	0.35 ± 0.07	0.26 ± 0.16
*Enterobacteriaceae unknown*	0.00 ± 0.00	0.03 ± 0.02
*F16 unknown*	0.02 ± 0.01	0.02 ± 0.01
*Anaeroplasma*	0.00 ± 0.00	0.09 ± 0.09
*ML615J-28 unknown*	0.00 ± 0.00	0.05 ± 0.01^*^
*Akkermansia*	0.43 ± 0.35	14.29 ± 1.86^***^
*Other*	0.16 ± 0.03	0.15 ± 0.07

Gray shading indicates tryptophan and nicotinamide metabolism associated microbiota.

^*^p < 0.05, ^**^p < 0.01, ^***^p < 0.001 when compared to C57BL/6 mice.

### 3.3 Differential expression of tryptophan and nicotinamide metabolism-associated genes in the *Winnie* mouse brain

#### 3.3.1 Genes and pathways related to tryptophan and nicotinamide metabolism were changed in *Winnie* mouse brains

**Figure 6:**
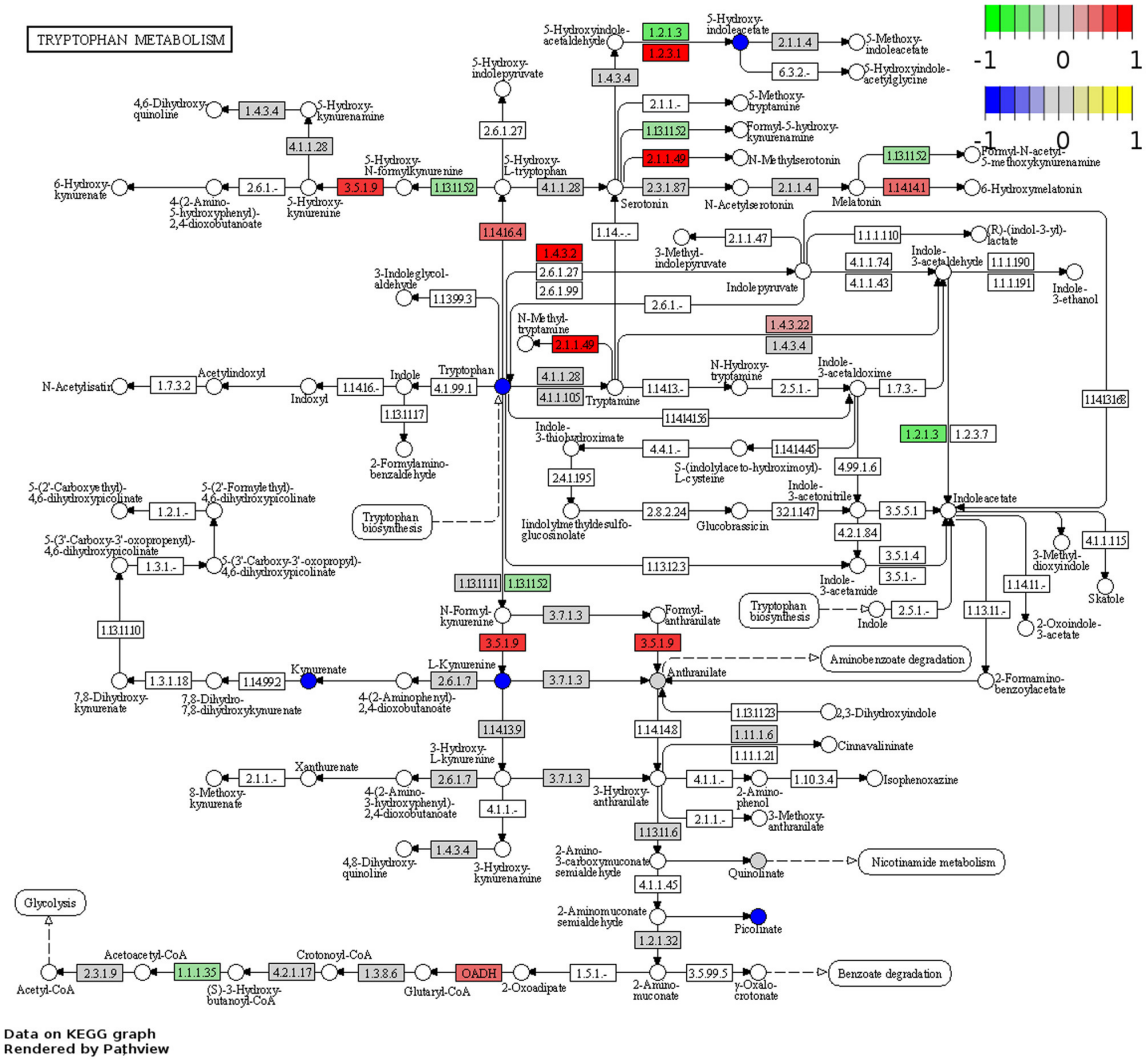
DEGs associated with tryptophan metabolism in the *Winnie* mouse brain. Pathview plot for the tryptophan metabolism KEGG pathway analysis of brain samples from *Winnie* mice (*n* = 6) vs. C57BL/6 littermates (*n* = 8). DEGs of the tryptophan metabolism KEGG pathways are colored according to their sign Log_2_FC × Log_10_*P* value changes. Rectangular nodes indicate gene expression data determined by RNA-seq. Positive values (red) indicate genes that are upregulated (tryptophan—EC:3.5.1.9, *Afmid*; EC:2.1.1.49, *SAMe*; EC:1.4.3.22, *Aoc1*; EC:1.14.16.4, *TpH1*; EC:1.4.3.2, *Laao*; EC:1.2.3.1, *Aox*; OADH, *Dhtkd1*; and EC:1.14.14.1, *Cyp1a1*, nicotinamide—EC:2.7.1.22 and EC:2.7.1.173, *Nmrk1*, and EC:1.2.3.1, *Aox*) and negative values (green) indicate genes that are downregulated (tryptophan—EC:1.13.11.52, *Ido1*, EC:1.2.1.3, *Aldh2*; EC:1.1.1.35, *Hadh*, nicotinamide—EC:2.4.2.1 *Pnp*; EC:3.2.2.6 and EC:249920, *Cd38*) in the *Winnie* mouse brain relative to brains from C57BL/6 mice. Circular nodes indicate the relative expression of tryptophan metabolism compounds.

**Figure 7:**
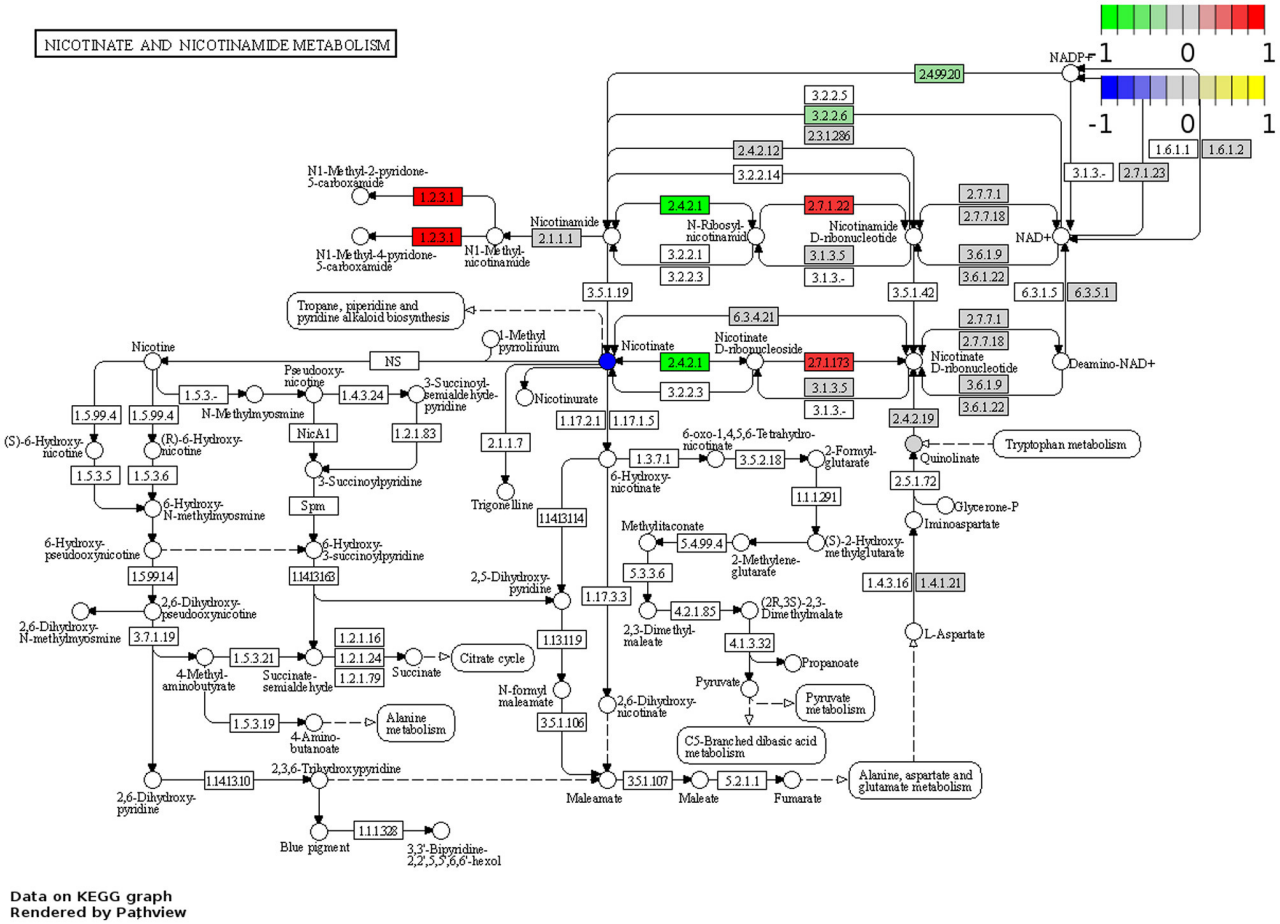
DEGs associated with nicotinamide metabolism in the *Winnie* mouse brain. Pathview plot for the nicotinate and nicotinamide metabolism KEGG pathway analysis of brain samples from *Winnie* mice (*n* = 6) vs. C57BL/6 littermates (*n* = 8). DEGs of the nicotinamide metabolism pathways are colored according to their sign Log_2_FC × Log_10_*P* value changes. Rectangular nodes indicate gene expression data determined by RNA-seq. Red represents upregulated gene expressions and green indicates genes that are downregulated in the *Winnie* mouse brain relative to brains from C57BL/6 mice. Circular nodes indicate the relative expression of nicotinamide metabolism compounds.

RNA-Seq was performed to determine changes in tryptophan and nicotinamide metabolism-associated gene expressions in the brains of *Winnie* mice (*n* = 6) vs. age-matched C57BL/6 littermates (*n* = 8). Visualization of the KEGG tryptophan and nicotinamide metabolism pathways showed changes to gene expressions in the *Winnie* mouse brain compared to brains from C57BL/6 mice (Figures 6, 7). Analysis of the transcriptome data in *Winnie* mouse brains revealed 11 DEGs associated with tryptophan metabolism compared to brains from C57BL/6 mice. Amine metabolic process was predisposed by the upregulation of *Afmid*, L-amino acid oxidase/monooxygenase (*Laao*), amine oxidase copper containing 1 (*Aoc1*), and cytochrome P450 family 1 subfamily A1 (*Cyp1a1*). Aromatic amino acid family metabolic and indole-containing compound processes were implicated by upregulated L-tryptophan hydroxylase (*Tph1*), *Afmid*, and *Laao*. The upregulated *Afmid* and *Laao* are involved in the tryptophan catabolic process, whereas upregulated *Tph1, Afmid*, dehydrogenase E1 and transketolase domain containing 1 (*Dhtkd1*), *Laao*, and *Cyp1a1* affect carboxylic acid metabolic process in fatty acid pathways. The downregulation of *Ido1* associates with the conversion of tryptophan to kynurenine. Both hydroxyacyl-CoA dehydrogenase (*Hadh*) and aldehyde dehydrogenase (NAD^+^) (*Aldh2*) are involved in the degradation pathways of fatty acids and branched-chain amino acids (valine, leucine, and isoleucine). Downregulation of *Ido1, Hadh*, and *Aldh2* suggested a decrease in metabolic capacity and impacted oxidoreductase activity, affecting redox-sensitive signaling pathways. There were four DEGs associated with the nicotinamide metabolism pathway in the *Winnie* mouse brains when compared to brains from C57BL/6 mice. Aldehyde oxidase (*Aox1*) and *Nmrk1* were upregulated, enhancing broader metabolic processes, while downregulated *Pnp* indicates impaired pyrimidine and purine metabolism.

#### 3.3.2 Changes in concentration of tryptophan metabolites in the *Winnie* mouse brain

**Figure 8:**
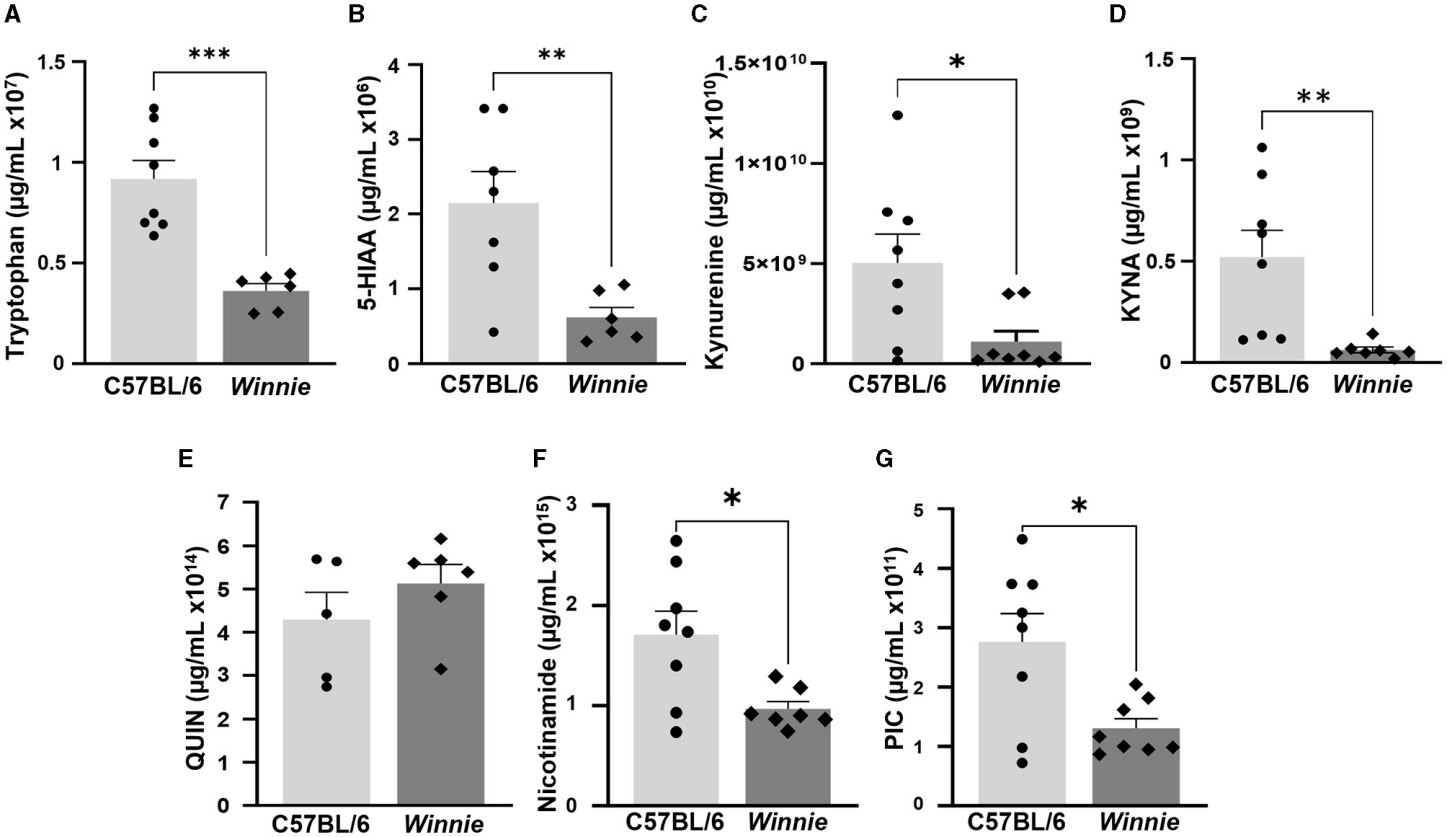
Changes in tryptophan metabolite concentrations in *Winnie* mouse brains. Quantification of tryptophan metabolites (μg/ml) measured by UFLC in *Winnie* mouse brains compared to brains from C57BL/6 mice (*n* = 5–8 for both). **(A)** Tryptophan, **(B)** 5-HIAA, **(C)** Kynurenine, **(D)** KYNA, **(E)** QUIN, **(F)** Nicotinamide, and **(G)** PIC. Data is expressed as mean ± SEM, *p < 0.05, **p < 0.01, ***p < 0.001 when compared to C57BL/6 mice.

Metabolomics analysis was used to quantify tryptophan metabolite concentrations, including tryptophan, 5-HIAA, KYNA, kynurenine, QUIN, nicotinamide, and PIC, in the brains of *Winnie* mice and compared to brain samples from C57BL/6 littermates (*n* = 5–8 for both, Table 3, Figure 8). In the *Winnie* mouse brains, there were significantly lower concentrations of tryptophan (*p* < 0.001), 5-HIAA (*p* < 0.01), kynurenine (*p* < 0.05), KYNA (*p* < 0.01), PIC (*p* < 0.05), and nicotinamide (*p* < 0.05) when compared to brain samples from C57BL/6 mice. There was no difference in the concentration of QUIN between groups (Table 3, Figure 8). Changes in the concentration levels of tryptophan metabolites are reflected in changes in the expression of genes involved in these pathways.

**Table 3:** Tryptophan metabolite concentrations in the brains from C57BL/6 and *Winnie* mice.

**Metabolite**	**Concentration (**μ**g/ml)**	
	**C57BL/6**	***Winnie***
Tryptophan	9.18 × 10^6^ ± 0.91 × 10^6^, *n* = 8	3.62 × 10^6^ ± 0.36 × 10^6^^***^, *n* = 6
5-hydroxyindole-acetic acid (5-HIAA)	21.48 × 10^5^ ± 4.19 × 10^5^, *n* = 7	6.19 × 10^5^ ± 1.33 × 10^5^^**^, *n* = 6
Kynurenine	5.05 × 10^9^ ± 1.43 × 10^9^, *n* = 8	1.11 × 10^9^ ± 0.53 × 10^9^^*^, *n* = 8
Kynurenic acid (KYNA)	5.22 × 10^8^ ± 1.32 × 10^8^, *n* = 8	0.64 × 10^8^ ± 0.15 × 10^8^^**^, *n* = 7
Quinolinic acid (QUIN)	4.29 × 10^14^ ± 0.63 × 10^14^, *n* = 5	5.13 × 10^14^ ± 0.63 × 10^14^, *n* = 6
Nicotinamide	17.08 × 10^14^ ± 2.37 × 10^14^, *n* = 8	9.66 × 10^14^ ± 0.74 × 10^14^^*^, *n* = 7
Picolinic acid (PIC)	2.76 × 10^11^ ± 0.48 × 10^11^, *n* = 8	1.31 × 10^11^ ± 0.16 × 10^11^^*^, *n* = 8

^*^p < 0.05, ^**^p < 0.01, ^***^p < 0.001 when compared to C57BL/6 mice.

## 4 Discussion

In the current study, we used *Winnie* mice, an experimental model of spontaneous chronic colitis established as highly representative of IBD, to identify changes in tryptophan and nicotinamide metabolism-associated genes and metabolites in the colon and brain, as well as explore alterations in the abundance of fecal microbiota involved in tryptophan metabolism. Our results demonstrated dysregulated tryptophan metabolism and *de novo* NAD^+^ synthesis corresponding to dysbiosis of the microbiota, indicating disruptions to the gut-brain axis associated with chronic intestinal inflammation.

Consistent with our findings of upregulated *Ido1* expression in the *Winnie* mouse colon, IDO1 is increased in tissues from IBD patients and other animal models of colitis in response to inflammation (68–72). Upregulation of *Ido1* corresponds to increased concentrations of tryptophan and kynurenine in colons from *Winnie* mice in this study, given that IDO1 is the first and rate-limiting step in tryptophan catabolism along the kynurenine pathway (20). Overexpression of IDO1 and subsequent increased kynurenine levels are implicated in inhibiting reactive effector T and natural killer (NK) cell responses, which are dysregulated in *Winnie* mice (37, 73). Previous studies have shown that species belonging to *Lactobacillus* and *Bifidobacterium* may shift the host tryptophan metabolism by suppressing the kynurenine pathway (24, 74). Specifically, *Lactobacillus* can reduce IDO1 activity in intestinal epithelial cells and decrease the production of kynurenine compounds (75, 76). Therefore, a reduction in the abundance of *Lactobacillus* and *Bifidobacterium* in fecal samples from *Winnie* mice in this study may be correlated to the upregulation of *Ido1* and increased kynurenine in the distal colon. It is widely accepted that the kynurenine metabolism pathway of IBD patients is shifted toward QUIN compared to the healthy population (15). This suggests that the upregulation of *Kynu* observed in *Winnie* mice colons in this study promoted the conversion of kynurenine toward QUIN, a neurotoxic metabolite, which was correspondingly increased in the colons from *Winnie* mice. QUIN can increase the expression of *N*-methyl D-aspartate receptor 2B in the ENS and, in turn, leads to intestinal symptoms and mood disorders associated with gut inflammation (77). Furthermore, increased QUIN may be correlated with disease severity and a pro-inflammatory environment in the *Winnie* mouse colon, as previously demonstrated in mice with DSS-induced colitis (78). Although gut microbial phyla, such as *Firmicutes* and *Bacteroidetes*, contain organisms that encode for the *Kynu* gene (79), further meta-transcriptomic studies are required to determine any association between changes in microbial abundance and upregulation of *Kynu* in the *Winnie* mouse colon observed in this study.

Differential expression of several tryptophan metabolism-associated genes in the *Winnie* mouse colon, is also associated with oxidative stress, a pathophysiologic factor implicated in colonic inflammation in patients with IBD and *Winnie* mice (41, 80). For instance, *Mao-b*, involved in the production of hydrogen peroxide, was upregulated in the *Winnie* mouse, consistent with increased levels of the *Mao* substrate, tyramine, previously reported in fecal samples from *Winnie* mice (40). Elevated levels of tyramine and 5-HIAA are known to affect GI dysmotility (81, 82). In this study, the concentration of 5-HIAA was increased in the *Winnie* mouse colon, corresponding to upregulated *Mao-b* expression, as well as altered colonic function and impaired smooth muscle contractility previously shown in this model (35, 39). Additionally, our finding of downregulated *Afmid* in the *Winnie* mouse colon is supported by increased aldehyde, butanal, and propanol in *Winnie* mice feces (40). This aberration may be initiated by the upregulation of *Mao-b* and *Aldh2* expression described in this study, thereby increasing the susceptibility of the colon to aldehyde-induced tissue damage (83). Correspondingly, our finding of downregulated *Dhtkd1* may be associated with impaired mitochondrial function and increased reactive oxygen species (ROS) production, which have been previously described in the *Winnie* mouse colon (41, 84). Although levels of tryptophan are generally proportional to the expression of *Dhtkd1* (85), the tryptophan concentration in the *Winnie* mouse colon was increased. Nicotinate interacts with the tryptophan pathway of *de novo* NAD^+^ biosynthesis from QUIN, which was also increased in *Winnie* mice colons in this study. Since this pathway is an alternative to the tryptophan degradation to 2-oxoadipate, reduced *Dhtkd1* expression may increase the tryptophan flux to NAD^+^ synthesis (85).

In this study, the downregulation of S-adenosyl-L-methionine (*SAMe*) in the *Winnie* mouse colon may suggest an enhanced bacterial production of anionic sulfide, culminating in acute oxidative damage (86, 87). Alterations in sulfur metabolism and subsequent overproduction of hydrogen sulfide can be driven by *Akkermansia*, which were increased in fecal samples from *Winnie* mice in this study (88). Furthermore, reduced *Bifidobacteria, [Prevotella]*, and *Lactobacillus* may correspond to the downregulation of *SAMe* in the *Winnie* mouse colon as decreased folate, butyrate, and acetate-producing microbiota can induce changes in host cell DNA methylation patterns via altered synthesis of epigenetically active metabolites necessary for generation of SAMe (89). Downregulation of L-tryptophan decarboxylase (*Ddc*) is reported to constrain the enzymatic activity of GI microbes, causing an imbalance in intestinal microbiome composition (62). Importantly, the synthesis of tryptamine is catalyzed by *Ddc* expressed by gut microbiota, such as members of the *Lactobacillus, Clostridia*, and *Ruminococcus* genera (63). In this study, downregulated *Ddc* may be associated with changes in the abundance of these bacteria in fecal samples from *Winnie* mice, reflecting disrupted tryptamine synthesis. However, further association analyses are required to confirm potential correlations. Consistent with our findings of downregulated *Aoc1* in colons from *Winnie* mice, previous studies have reported decreased AOC1 in colon tissues from patients with UC (90, 91). Since decreased AOC1 diverts ornithine metabolism away from putrescine, our finding is substantiated by reduced putrescine levels previously reported in fecal samples from *Winnie* mice (40). Decreased diamine oxidase (DAO) activity is associated with changes to the abundance of *Bifidobacterium*, while certain strains of *Lactobacillus* are considered probiotic for DAO deficiency (92). Therefore, the downregulation of *Aoc1* in the *Winnie* mouse colon may be correlated with the reduced abundance of *Bifidobacterium* and *Lactobacillus* observed in this study.

Our study revealed differential expression of genes involved in nicotinate and nicotinamide metabolism in the distal colon of *Winnie* mice. The upregulation of *Nampt* is supported by previous studies reporting elevated Nampt in colon tissues from IBD patients and DSS-induced colitis mice counteracting an increased cellular NAD turnover mediated by NAD-depleting enzymes (93–95). *Nampt* upregulation initiates activation of the nuclear factor kappa light chain enhancer of activated B cells (NF-kB) signaling pathway in the IL-6 and TNF-α feedback loop, leading to an escalation in IDO1 activity (20). This correlates to the upregulation of *Ido1* and associated changes to microbial abundance observed in this study, thereby substantiating a mechanistic association between tryptophan catabolism and NAD^+^ synthesis in the *Winnie* mouse colon (96). Upregulation of *Art2a* amplifies the inflammatory/immune process (97). This contributes to depleted NAD^+^ availability, compromising mitochondrial function and triggering the release of ROS (98–100), consistent with the pathological conditions observed in *Winnie* mice (37). Furthermore, adenosine diphosphate (ADP)-ribosyltransferases are widely prevalent in the human gut microbiome and highly abundant in *Bacteroides* (101), thus the upregulation of ADP-ribosyltransferase 2a (*Art2a*) in the colon may be associated with the increased abundance of *Bacteroides* in fecal samples from *Winnie* mice in this study. Downregulation of *Nmrk1* and *Nmrk2* observed in the *Winnie* mouse colon may contribute to inflammation-associated ENS damage reported in a previous study (38), as in homeostatic conditions, *Nmrk* allosterically upregulates to safeguard neurons against axonopathy if NAD^+^ is depleted or under attack by NAD-consuming enzymes (102). Reduced expression of *Naprt* observed in our study can inhibit the production of NAD^+^ and prevent the protection of colon tissues from oxidative stress (103). The colitis in *Winnie* mice exhibits manifestations closely resembling IBD, such as tight junction damage, early intestinal cell death, and cellular stress responses (37, 39, 41, 104–109), which may be linked to glutamine deficiency. *Nadsyn1*, the gene encoding for a glutamate-dependent enzyme, was downregulated in *Winnie* mice, which suggests a response to glutamine deficiency (110). This notion is supported by a study in acute DSS-induced colitis mice that found glutamine sufficiency is required for the colonic epithelium to develop a cell-protective, antiapoptotic, and anti-inflammatory response to inflammatory damage (111).

Tryptophan metabolism is intricately linked with behavioral modulation, affecting mood, sleep, appetite, and cognitive function, therefore alterations in tryptophan metabolism-associated genes and tryptophan metabolites in the *Winnie* mouse brain demonstrate disruption of the gut-brain axis and may indicate behavioral changes associated with neurodegeneration and depression (25). Of significance, is the downregulation of *Ido1* and *Aldh2* in brains from *Winnie* mice, given IDO1's association with exploratory behavior, cognitive function, and depression (112–114), and *Aldh2*'s role in detoxifying ROS and ethanol metabolism, processes relevant to neurodegeneration (115, 116). The reduction in *Ido1* activity within the brain may serve as a protective mechanism to inhibit the over-degradation of tryptophan, an essential precursor to 5-HIAA (117). Since 5-HIAA was lower in *Winnie* mice brains, such regulation may ensure that serotonin levels remain sufficient to support standard brain functionality during systemic inflammation (112). In consistency with our findings, a reduced level of 5-HIAA in brains from mice with DSS-induced colitis is correlated with a decreased abundance of *[Prevotella]* and an increased abundance of *Ruminococcus*, indicating a close association between the gut microbiome and production of brain neurotransmitters (118). Additionally, *Laao1* upregulation in *Winnie* mice brains may alter the ratio of tryptophan to large neutral amino acids (LNAA) influencing serotonin synthesis (119). This is compounded by reduced concentrations of both tryptophan and 5-HIAA in the brains of *Winnie* mice in this study. The upregulation of *Tph1* in brains from *Winnie* mice is related to stress sensitivity and increased susceptibility to psychiatric pathologies (120), which are prevalent in IBD patients (121). Two of the dominant phyla, *Firmicutes* and *Proteobacteria*, both contain species with genes encoding *TPH1* (79) and *Lactobacillus* has been reported to promote butyrate production, ultimately stimulating TPH1 activity by butyrate and regulating serotonin synthesis (61). However, further studies are required to establish any correlations between the upregulation of *Tph1* and changes to the abundance of these microbiota in this study.

Differential expression of tryptophan metabolism-associated genes may indicate compensatory mechanisms with potential deleterious effects to preserve overall brain health in *Winnie* mice. Given that *Aldh2*, an enzyme involved in detoxification, is downregulated, the upregulation of *Afmid*, known to play a role in toxin elimination, could indicate action to restore some equilibrium to brain chemistry in *Winnie* mice (115, 122). Nonetheless, *Aox* was upregulated, indicating oxidative stress in the brains of *Winnie* mice. Plausibly, in an adaptive effort to optimize mitochondrial activity, *Winnie* mice allosterically upregulated *Dhtkd1*. However, variations in the *Dhtkd1* gene have the potential to disrupt tryptophan metabolism and the equilibrium of neuroactive metabolites, contributing to the onset of depression or other neurological disorders (84). Additionally, upregulated *SAMe* in *Winnie* mice brains may be indicative of disturbed biological rhythms, by inhibiting methylation processes (123).

Several factors contributing to the dysregulation of *de novo* NAD^+^ synthesis were identified in the *Winnie* mouse brains, including reduced nicotinamide metabolites. While the overall activity of NAD^+^-consuming enzymes remained largely unaltered, downregulated *Cd38* expression may be associated with lower levels of nicotinamide in *Winnie* in mice brains, potentially leading to behavioral changes previously observed in niacin deficient and *Cd38*^−/−^ mice (124). Reduced nicotinamide has been associated with depression, anxiety, and psychological stress, as well as contributing to neurodegeneration and compromising cellular defense mechanisms against stress and injury (125–127). Despite downregulated *Cd38*, the underlying cause of the reduced nicotinamide levels in *Winnie* mice brains remains unclear. However, lower levels of nicotinamide aligned with the upregulation of *Nmrk1*, an enzyme known to increase its activity in response to NAD^+^ depletion (128), and *Pnp* downregulation, a major component in nicotinamide riboside metabolism (129). Further investigations are required to elucidate the synthesis of NAD^+^ and its interplay with nicotinamide in *Winnie* mice brains.

Lower levels of tryptophan and kynurenine in *Winnie* mice brains can indicate disruption in transport mechanisms of these metabolites across the blood-brain barrier or failure to sufficiently compete with other large amino acids sharing the LNAA transporter (130). These findings are significant as the metabolism of tryptophan, via the kynurenine pathway, serves as a critical link between peripheral inflammation and central nervous system alterations (25). In contrast to our results, elevated kynurenine levels have been observed in humans with major depressive disorder (131), as well as in acute DSS-induced colitis mice (132). Acute inflammation in DSS-induced colitis stimulates the tryptophan-kynurenine-KYNA pathway in the brain (132); therefore, our findings suggest that chronic colitis in *Winnie* mice has a diminished impact on this pathway, given that neuroprotective KYNA concentration was reduced.

To further elucidate the complex interactions of tryptophan metabolism and its systemic effects in IBD, subsequent investigations should encompass a multifaceted approach. A detailed comparison of tryptophan concentrations in colonic tissue, plasma, and cerebral structures would provide a more holistic understanding of the metabolic disturbances characteristic of chronic intestinal inflammation. While RNA-Seq and metabolomics have provided a foundational descriptive analysis, indicating changes to the regulation of tryptophan and nicotinamide-associated genes and metabolites, there is a necessity to extend these findings through various biochemical and molecular techniques. Systems biology approaches could synthesize data from genomics, proteomics, and metabolomics to model the biological networks that underpin tryptophan metabolism, providing predictive insights and a systemic understanding of disease pathology. Furthermore, the application of multi-omic analysis could unravel complex interactions and feedback loops, giving a more comprehensive understanding of the disease mechanisms at play. Western blotting and mass spectrometry would enable the quantification and confirmation of the proteins of interest, potentially unveiling post-translational modifications and the impact of gene regulation on protein expression. Additionally, immunohistochemistry would allow for the localization of these proteins within tissue contexts, offering insight into the cellular and tissue-specific distribution and the pathological significance of these findings.

## 5 Conclusion

This study demonstrates alterations in tryptophan and nicotinamide metabolism-associated genes, microbiota, and metabolites in *Winnie* mice with spontaneous chronic intestinal inflammation. Our findings signify the role of the gut-brain axis in gut inflammation and further establish the *Winnie* mouse as an experimental model highly representative of human IBD. We report changes in gene expression that contribute to the shift of tryptophan metabolism toward the kynurenine pathway, impacting NAD^+^ synthesis and revealed the dualistic influence of 5-HIAA in intestinal health. These findings connect these metabolic pathways to behavioral, cognitive, and emotional challenges experienced by IBD patients and suggest that tryptophan metabolism may exert a greater influence on the kynurenine and serotonin pathway than previously recognized. The insights gathered from this study, along with forthcoming data, can be utilized to enhance the identification and management of IBD through the development of targeted therapeutic interventions. Furthermore, these findings provide a compelling foundation for undertaking further detailed investigations into the association between intestinal inflammation, tryptophan metabolism, and behavior.

## Data availability statement

The datasets presented in this study can be found in online repositories. The names of the repository/repositories and accession number(s) can be found at: https://www.ncbi.nlm.nih.gov/geo/, GSE244558 (colon data), GSE264317 (for brain data) and https://github.com/Nurgali-lab/C57-Win-APX-16SV3-4.git, C57-Win-APX-16SV3-4.

## Ethics statement

The animal study was approved by Victoria University Animal Experimentation Ethics Committee. The study was conducted in accordance with the local legislation and institutional requirements.

## Author contributions

JD: Conceptualization, Data curation, Formal analysis, Writing – original draft, Writing – review & editing. AR: Data curation, Formal analysis, Writing – original draft, Writing – review & editing. RS: Formal analysis, Writing – review & editing. MD: Data curation, Formal analysis, Writing – review & editing. ND: Data curation, Formal analysis, Writing – review & editing. RE: Data curation, Formal analysis, Writing – review & editing. DK: Supervision, Writing – review & editing. VA: Supervision, Writing – review & editing. KN: Conceptualization, Project administration, Resources, Supervision, Writing – review & editing.

## Supplementary material

Supplementary Figure S1 Heat map representation of gene lists for tryptophan and nicotinamide-associated gene expressions. Gene lists for tryptophan and nicotinamide-associated gene expressions were generated by merging related gene lists from multiple databases accessed via the molecular signature database (MSigDB).
