# Supplementary figures and images for: Alterations in tryptophan metabolism and de novo NAD+ biosynthesis within the microbiota-gut-brain axis in chronic intestinal inflammation
Source: Front Med (Lausanne). 2024 Jul 2;11:1379335. doi: 10.3389/fmed.2024.1379335 (PMC11250461; doi:10.3389/fmed.2024.1379335)

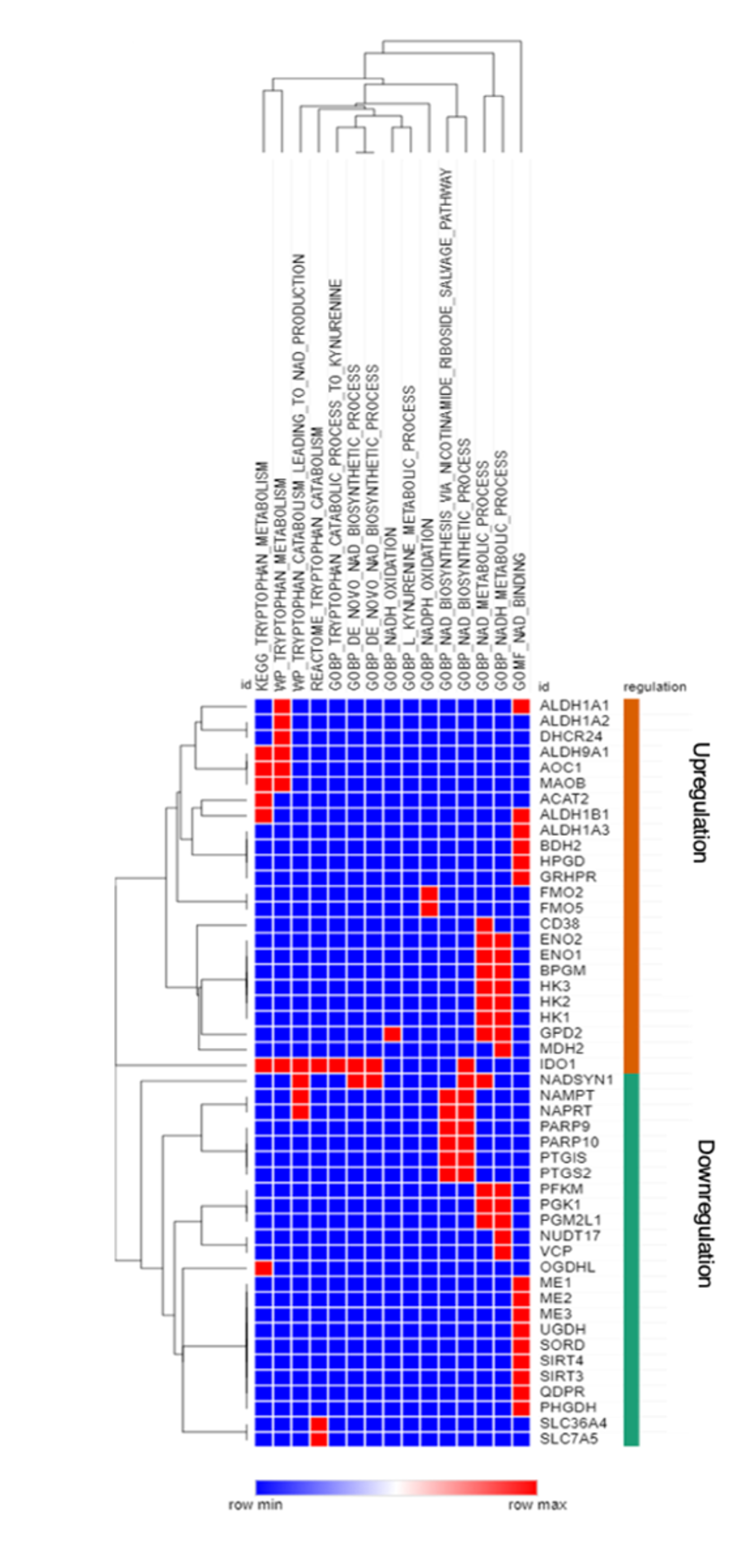

Supplement: Supplementary Figure S1 — Heat map representation of gene lists for tryptophan and nicotinamide-associated gene expressions. Gene lists for tryptophan and nicotinamide-associated gene expressions were generated by merging related gene lists from multiple databases accessed via the molecular signature database (MSigDB). [file Image_1.TIF]
